# Meat and social change: Sociological perspectives on the consumption and production of animals

**DOI:** 10.1007/s11614-021-00453-0

**Published:** 2021-07-07

**Authors:** Frithjof Nungesser, Martin Winter

**Affiliations:** 1 Austrian Academy of Sciences | Department of Sociology, University of Graz, Universitätsstraße 15/G4, 8010 Graz, Austria; 2 Department of Sociology, Technical University of Darmstadt, Dolivostraße 15, 64293 Darmstadt, Germany

## The sociological significance of meat

Meat is a crucial object of sociological research. The *consumption* of meat plays a significant role in the food supply of modern societies. The importance of meat is not, however, limited to its nutritional value. Rather, the preparation and consumption of meat dishes is linked to cultural traditions and norms, collective and individual identities, as well as to gender relations and conceptions of health, purity, or naturalness. The *production* of meat is connected to numerous ecological problems, the breeding and killing of billions of animals, precarious working conditions and issues of public health (as, for example, the current pandemic demonstrates). Moreover, both the consumption and production of meat are linked to various *dynamics of transformation and conflict*: Technical and scientific innovations as well as political and economic decisions transform agriculture and meat production, leading to an unparalleled productivity but also to unprecedented environmental consequences. While the “normality” of meat consumption spreads further around the globe and is no longer exclusive to the Global North, in Western societies problems related to meat increasingly become the subject of public debates and social struggles.

Despite the diverse and far-reaching implications and consequences of meat production and consumption, the sociological debate on meat is rather new. At first sight this may be surprising since important aspects of meat production and consumption are addressed by classical sociological authors. Norbert Elias ([1939] 2000, p. 100), for example, states that “[p]eople’s attitudes to meat-eating […] are highly illuminating with regard to the dynamics of human relationships and personality structures.” Accordingly, the changing relationship of different social classes to the killing of animals as well as to the preparation and consumption of meat figures largely in Elias’s analyses of the “civilizing process”. Also, in his seminal study *Distinction*, Pierre Bourdieu ([1979] 1984, pp. 183–193) sheds light on the distinctive character of specific kinds of meat and highlights the crucial link between meat consumption, class and gender. Such classical insights notwithstanding, sociology neglected the agro-food-sector as a research topic for a long time. It is only in recent years that an increasing sociological attention to the subject of food and nutrition has emerged (cf. Paulitz and Winter 2019). In the wake of the increased sociological engagement with food, several relevant works dedicated to the topic of meat have been published (e.g. most recently Rückert-John and Kröger (2019); see *Rose* in this volume for a review).

Why did it take so long to perceive food and eating in general as well as meat in particular as important sociological research topics? In our view, two main reasons can be identified—at least with regard to the German speaking discourse. The first reason is that, for a long time, food, eating and nutrition were predominantly conceived as biological phenomena that have to be studied by the natural sciences. However, scientific approaches focus on the “biological needs” of bodies and tend to ignore the social, cultural and ecological dimensions of food and eating.^1^ Accordingly, the questions of how people feed their bodies and how food is processed by the body are seen as a matter of “nature” that do not interfere with social and cultural processes (cf. Paulitz and Winter forthcoming). In contrast, in the first major works in the sociology of eating, published in the 1990s, food was conceptualized as both a natural as well as a social and cultural phenomenon (Barlösius 1993, [1999] 2011; Wierlacher et al. 1993). Eva Barlösius’ groundbreaking insights offer a theoretical framework to understand the relation between food, body and society and open up the nature/culture-divide for sociological debate (Paulitz and Winter 2017). Especially with regard to meat, the significance of such a sociological perspective on nutritional knowledge becomes evident. For example, both in past and present times meat-free diets have often been met with skepticism because of a kind of nutritional folk knowledge that understands meat as indispensable to a balanced diet and a healthy body. Only from a sociological viewpoint it becomes obvious that discussions like these are not only about the correct calculation of nutritional values or physiological needs. Rather, nutritional knowledge as well as food-related debates are themselves connected to specific cultural ideas about naturalness, health or gender and thus to social hierarchies and power relations.

The second reason is that food and nutrition quite generally have become objects of increased attention both in public discourse and in everyday-life. This increase in attention corresponds with marked changes in dietary patterns, the diffusion and break-up of culinary traditions and the flourishing of alternative diets.^2^ Over time, practices of food preparation and consumption lost their self-evident character and the “modern subject of food choice” (Coveney 2006) emerged. From a sociological point of view, this changed relationship to food can be interpreted as a result of broader transformations of society. Quite generally, it can be traced back to the pluralization and individualization of lifestyles that made it possible to distance oneself from culinary traditions (Jallinoja et al. 2019, p. 160). A more reflexive relationship to food consumption was also fostered by the rise of postmaterialist values and consumerism. In the last decades, at least in certain milieus, moral or political worldviews were increasingly translated into “non-electoral political acts” (Copeland 2014, p. 258) such as the decision to buy and consume only certain kinds of food (Jallinoja et al. 2019; Lorenz 2006). Furthermore, the changes in eating habits can be understood as an example for the “neoliberal” transfer of social risks from the collective to the individual (Lessenich 2008). Within a neoliberal framework, the responsibility for the consequences of agricultural and livestock production are attributed to the consumption behavior of individuals. To live in a healthy, eco- and animal-friendly way, thus, becomes a matter of individual responsibility and risk management (Brunner 2011, p. 203; Winter 2019). The continuing trend of vegetarian and vegan diets is the most notable development within the field of nutritional change. Nutritionists and experts for vegetarian lifestyles Christoph Leitzmann and Markus Keller (2010, p. 17) report that in the 1980s less than 1% of the German population declared themselves as vegetarian. In Germany today, following the latest Nutrition Report (“Ernährungsbericht”, BMEL 2019, p. 5), around 6% of the German population are vegetarian, current market research data even suggest a proportion of 9.2% vegetarians and 1.6% vegans (IfD Allensbach 2020, p. 81). Additionally, a new consumer-category between and beyond the rigid definitions of vegetarianism and veganism evolved: flexitarianism (cf. Kofahl and Weyand 2016).

As these remarks show, in the last years different developments within the social sciences, public discourse and every-day life pointed to the sociological significance of meat. Three aspects are of special importance in this respect: First, it became clear that the consumption of meat is not “naturally” predetermined but socially and culturally induced, regulated and restricted. Second, it became apparent that the production of meat has to be understood as situated in culture-specific human-animal relations as well as in political and economic frameworks. Third, it became obvious that, over time, meat consumption and production have been transformed into controversial practices that are a crucial element of political, moral and environmental struggles. In the following three sections, we briefly outline each of the aspects that we address as the “three Ds” of meat: distinction, domination, and dissonance. We discuss how the consumption (Sect. 2) and production (Sect. 3) of meat as well as the struggle over meat (Sect. 4) are related to central driving forces of social change. As will become clear, the articles assembled in this issue relate to the developments described and contribute to a better understanding of all three aspects of the social significance of meat.

## Distinction: the consumption of meat

To shed light on the social significance of meat consumption, *the meal* is a good starting point. The importance of the meal as a social institution is emphasized by Georg Simmel ([1910] 1997) in a classical essay. As Simmel argues, the meal cannot be reduced to the satisfaction of biological needs. Rather, as he shows, eating together connects the nourishing of the body with processes of social integration and the aestheticization of every-day practices. Simmel ([1910] 1997, p. 131) distinguishes between “food as substance” and the “form of its consumption.” He then focusses on the formal aspects and describes how, especially in “educated circles,” the meal becomes a highly aestheticized activity that is “schematized and regulated on a supra-individual level” (Simmel [1910] 1997, p. 132). As Mary Douglas (1972) shows in another classical study, the sociological perspective on food is not limited to its formal and aesthetic dimensions. In her structuralist analysis, she decodes the composition of the meal itself. She coins the formula of a meal as “a + 2b”, where “a is the stressed item and b the unstressed item” (Douglas 1972, p. 68). Meat typically is of category “a.” While Douglas does not equate category “a” with meat, anthropologist Nick Fiddes (1991, p. 14) describes how only a meat-containing meal is commonly considered as “proper food.” Moreover, as already Simmel ([1910] 1997, p. 131) noted, the meal mirrors the hierarchy of the table fellowship (see also Symons 1994, p. 347). Monika Setzwein (2004, p. 213) remarks that within families the (commonly male) head of family is typically served first and the best piece of the roast. Thus, meat must be regarded not only as the central ingredient in contemporary diets but also as a crucial element in distinctive food practices: the total amount and the types of meat as well as the manner of preparation and consumption differ between and differentiate social groups.

The consumption of meat and its distinctive power are closely *entwined with the emergence and development of capitalist and industrialized society*. Drawing on slaughter statistics, German social historian Hans Jürgen Teuteberg (1988, p. 73) shows that meat consumption increased “surprisingly parallel to industrialization”.^3^ Before the rise of capitalism, Norbert Elias ([1939] 2000, p. 100) argues, meat consumption was reserved to the secular upper class, which ate “extraordinarily high” amounts of meat. In contrast, the clergy, especially in the monasteries, refrained from eating meat, decrying the quantity of meat consumption in the secular classes as gluttony. The medieval lower classes, again, suffered shortage of food in general. Thus, meat was a distinctive food before capitalism, but its role then changed fundamentally. In the course of the 19th century, the *discipline of nutritional science* gained importance as a social institution for the transmission of food-related knowledge. Two aspects are of special importance in this respect. First, as Ole Fischer (2015, p. 49–55) shows, the conception of meat changed significantly: As nutritional knowledge shifted towards a biochemical model, meat was no longer considered as difficult to digest but as healthy and nourishing food. Moreover, due to its amount of protein, meat was now seen as necessary for muscle growth. This, in turn, led to a masculine connotation of meat, because physical strength was especially seen as a necessity for laboring men. Second, nutritional science was biopolitically mobilized to calm social conflict. Nutritional knowledge was used to calculate food rations (“Kostsätze”) to scientifically justify food supply for workers (Barlösius [1999] 2011, pp. 60–62). For example, nutritionist Carl Voit (1831–1908) advised that especially workers should eat vast amounts of protein. To achieve this, meat was reserved to men in his guidelines (Mense 2007, p. 24–25; Pfister and Staub 2006, p. 5). Hence, the general rise in meat consumption is tied to the establishment of a capitalist class society and the assignment of hard manual labor to men. In industrialized societies meat is then transformed into a mass-commodity and the masculine connotation of meat is culturally perpetuated and persists (Winter 2018).

Only against the background of the increased availability and affordability of meat, Bourdieu’s well-known observations of different food tastes and practices can be understood. In his study of the French society of the 1960s, Bourdieu ([1979] 1984, pp. 175–193) compares the *consumption patterns of different classes and class fractions*. Based on this comparison he juxtaposes a “taste of necessity” and a “taste of luxury.” Whereas the “taste of necessity” is characteristic of the dominated classes that prefer “heavy, fatty, fattening foods” (Bourdieu [1979] 1984, p. 177), the “taste of luxury,” typical of the dominant classes, focusses not only on the form and manner of consumption but also on different foods. As Bourdieu and other sociologists show, this differentiation of tastes applies especially to meat, which is closely connected to both class and gender. Building on Bourdieu’s work, Petra Frerichs and Margareta Steinrücke (1997, p. 252) argue in a comparative cross-class interview study that meat as “masculine power food” is a “separation mark” between proletarian and other consumers. According to such a Bourdieusian view, members of the lower classes prefer coarse and fat types of meat such as pork, whereas members of the upper classes favor “leaner, lighter (more digestible), non-fattening” (Bourdieu [1979] 1984, p. 177) kinds of meat such as veal, lamb, mutton and especially fish, which Bourdieu ([1979] 1984, p. 190) interprets as utterly ‘unproletarian.’ Thus, similar to historical studies, Bourdieu underscores the *link between meat, masculinity and hard work*: “Meat, the nourishing food par excellence, strong and strong-making, giving vigour, blood, and health, is the dish for the men, who take a second helping, whereas the women are satisfied with a small portion.” (Bourdieu [1979] 1984, p. 192) Importantly, however, in the middle and upper strata of society Bourdieu identifies important differences within the classes. The class fractions in which economic capital dominates tend to live life to the fullest, while those fractions richer in cultural capital cultivate an “ascetic” element in their lifestyle, which often includes a “controlled diet” (Bourdieu [1979] 1984, p. 213). Here, Bourdieu touches on another crucial dimension of meat consumption that he does not elaborate on, however. Bourdieu only briefly mentions vegetarianism as one element of “counter-culture” and interprets it as a “desperate effort to defy the gravity of the social field” (Bourdieu [1979] 1984, p. 370).

In order to integrate meat-free lifestyles systematically into a Bourdieusian framework, Barlösius ([1999] 2011, pp. 117–118) adds a *third taste* to Bourdieu’s dualism. The “natural eating style,” she claims, is characterized by a *strong ascetic orientation and is based on moral imperatives*. Building on this thesis, abstinence from meat consumption can also be seen as a mode of distinction (Jallinoja et al. 2019, p. 170). Processes of ascetic, moral or “ecological distinction” (Neckel 2018) are used to explain that today at least certain milieus of the middle and upper classes consume significantly less meat than less privileged classes.^4^ Further, in multiple contemporary discourses meat consumption is questioned because of its ethical, ecological and health-related consequences (Jallinoja et al. 2019, pp. 158–160). Against this background, the question arises how meat-centered consumption patterns could be changed. Due to the strong symbolic ties of meat, this involves complex strategies to interfere with food practices (Rückert-John 2017). In her contribution to this issue, *Laura Einhorn* critically discusses the literature dealing with the relationship between class and meat consumption and the likeliness to change one’s diet. So far, she claims, research has mainly focussed on the statistical correlations between meat consumption and class. Little attention has been paid, however, to the question of how class differences could explain varying inclinations and opportunities for dietary *change*. Drawing on multiple qualitative interviews, Einhorn shows that the transition to a meat-free diet relies heavily on financial as well as non-financial resources that are unevenly distributed. Higher classes are thus more likely to change their diets.

Meat is not only perceived as a masculine food because of its association of being necessary for muscle formation. In her comprehensive study on the symbolic meanings of meat, Carol Adams ([1990] 2010) argues that *patriarchic dominance* is strongly linked to the *human rule over animals*. She describes semantic analogies between violence against women and violence against animals, which becomes especially obvious in sexualized advertisements for meat (see also Trittelvitz 2020). Regarding masculinity a more complex view of its relationship to meat is needed: Different attitudes towards meat vary, for example, between different constructions of masculinity that are ordered hierarchically in relation to each other. Jeffery Sobal (2005) proposes to differentiate the “strong man”, who eats a lot of meat to gain muscle strength, the “healthy man,” who reduces meat consumption, and the “wealthy man,” who consumes high status meat. In addition, Winter (2019) describes a “compassionate masculinity” for vegan eating styles. While the persistence of meat’s masculine connotation is well researched, *Ricarda Kramer* (in this issue) can show that the reduced meat consumption of women (as compared to men), that Bourdieu only touched on, is regulated via the connotation of being a risk for the health and attractiveness of the female body. In a qualitative analysis of women’s magazines, she shows how not only the amount but also the types of meat are regulated: Women should, following the discourse in these magazines, preferably eat meat that is considered to be healthy and lean, such as poultry or fish, and restrain from red meat.

Since most research focusses on the relations and intersections of meat consumption to class and gender, from our perspective, further research is required to understand how *other categories of social differentiation and inequality*, such as ethnicity or generation and age, relate to meat. In this vein, Larissa Deppisch (2019) describes how pork has become a symbol of anti-Muslim racist propaganda in Europe. Conservative and (far-)right-wing politicians demand the right to eat pork in public canteens, despite the fact that it has never been contested. Other research studies the effects of “ethnic food shops” on their urban environments. Shops that offer vegetarian meat alternatives such as falafel, it is argued, can be described as a driving factor of gentrification, since they attract customers with high cultural capital (Stock and Schmiz 2019). Publications like these demonstrate how the consumption of meat and meat alternatives interrelate with a broad spectrum of sociodemographic differentiations and inequalities.

## Domination: the production of meat

Sociological perspectives also contribute to a better and more nuanced understanding of the production of meat. We identify three strands of research that are of particular importance.

First, livestock breeding and meat production are crucial aspects within the broader context of *culture-specific human-animal relations* which, in turn, are shaped by specific cultural and social histories. That human collectives relate to other species in fundamentally different ways has been a focal point of recent discussions in cultural anthropology. In his pathbreaking study *Beyond Nature and Culture*, French anthropologist Philippe Descola ([2005] 2014) argues that all collectives organize their relations to other species according to one of four ontologies: naturalism, analogism, animisms or totemism. According to Descola, in Western societies, human-animal relations are organized by a naturalist ontology. On the one hand, such an ontology is characterized by the fact that other beings are perceived as similar with respect to their outward appearance and bodily nature—that is, with respect to their “physicalities.” On the other hand, with respect to feelings, subjectivity or reflexivity—that is, with respect to the “interiorities”—the Western naturalist ontology conceptualizes humans as drastically different from other beings (Descola [2005] 2014, ch. 3). The naturalist ontology establishes a strict hierarchy between different kinds of organisms, which has found expression in various theories and metaphors such as the century-old notion of the “great chain of being” (Lovejoy 1936) or the more recent evolutionary metaphor of the “tree of life”. Within such a hierarchical ontological framework, the use, killing and consumption of animals is easier to legitimize and is less in need of ritual compensation than, for example, within an animistic ontology, which Descola studied in Amazonia. However, as Descola himself shows, the Western naturalist ontology was always characterized by tensions between strictly dualist and more gradualistic positions. Already Descartes’ dualist view can be contrasted with Montaigne’s gradualism. In recent decades, social movements like the animal rights movements or academic developments like cognitive ethology or the recently emerging field of human-animal studies challenge dualist conceptions and criticize the treatment of animals in Western societies. From Descola’s viewpoint, these developments (and also the articles included in this issue) can be seen as expressions and interpretations of the tensions within the naturalist ontology.

Another way to make sense of the social and cultural constitution and perpetuation of the hierarchical and violent relation of humans and animals is to interpret it in terms of “ideology.” So far, such an approach has been used especially in social psychological studies that try to explain how individuals legitimize the killing of animals for food. Social psychologist Melanie Joy prominently coined the term “carnism” to identify an “ideology” that conceives of eating meat as “normal, natural and necessary” (Joy 2010, pp. 30, 96). Because individuals acquire a “carnist” set of schemas in the course of their socialization, Joy (2010, p. 18) argues, they learn to empathize with certain classes of animals (especially pets) but become “numb” to the suffering of other classes (especially livestock), thus avoiding “moral discomfort”. In this issue, *Christian Stache *and* Christin Bernhold*, present a different interpretation of the ideological underpinnings of meat production. Drawing on the work of Antonio Gramsci, they argue that the ideologies that legitimize eating meat need to be seen as embedded in a capitalist class society. Within such a society a “meat hegemony” prevails and stabilizes the domination of the ruling class and especially the meat capital over subaltern classes as well as over animals.

Second, perspectives that focus more on the *material object of meat* can be found in the area of Science and Technology Studies. Quite generally, the materiality of meat poses major challenges for social theory. To address the ambivalence of livestock animals between a living being and an agro-industrial product, philosopher of technology Nicole C. Karafyllis (2003, 2008) proposes the portmanteau word “biofact” that blends the words “biotic” and “artefact”. Following Karafyllis, biofacts are objects that lie between the natural and the artificial. This concept is taken up by Tanja Paulitz and Martin Winter (2018, pp. 14–15) in order to analyze the production of meat. They argue that the innovation, design and production of meat products has to be analyzed as interwoven with the construction of masculinity: They propose an inseparable relationship between the embodying of the already discussed ideal of the strong and muscular male body and the production of meat products that support this ideal, for example by increasing and/or stressing its amount of protein. Hence, they conceive food, bodies and gender as a co-production, thus emphasizing the connectedness of production and consumption.

Undermining the nature/culture divide more radically and discussing the above outlined theory of Descola, Emily Yates-Doerr and Annemarie Mol (2012) state that also in Western cultures, meat and animals cannot be found in one singular ontological form. Drawing on an actor-network approach that explores the material reality of things and considers these realities as multiple enactments, Yates-Doerr and Mol (2012, p. 50) argue that meat is “done differently” in the practices of a butcher, in preparing meat dishes in haute cuisine restaurants and in nutrition classes. In each context meat is enacted as a different object with different properties. With this micro-sociological approach, the authors claim that the very material reality of things is enacted in different practices in a different way. Food in different practices is “simply not the same thing” (Mol 2013, p. 381). Thus, what meat *is, *is not pre-socially given, but enacted in practice.

Third, sociological studies are also important with regard to the concrete *material and economic organization of animal production and processing*. From a historical perspective, we can identify an enormous increase in the number of animals killed in the meat industry (Nungesser 2018, pp. 164–166). Just as American sociology, the industrialization of animal violence started in the second half of the 19th century in Chicago and it intensified enormously in 1950s and 1960s when factory farming became the dominant way of livestock breeding (Fitzgerald 2010, pp. 60–62; Horowitz 2006). According to the Food and Agricultural Organization (FAO), the global number of livestock slaughtered per year increased by 783% in the last 55 years—from 8.4 billion in 1961 to 74.2 billion in 2016.^5^ In the same time span the world population increased by only 138% from 3.1 to 7.4 billion. Of course, the intensity of meat production and consumption differs significantly between world regions and countries. In the last years, the trend seems to be that in Western industrialized countries the numbers plateau on a high level, while in countries like China and especially India the numbers are drastically lower but rise quickly due to economic growth as well as cultural and social transformations. Hence, the numbers just presented will continue to increase (FAO 2006, pp. 6–9, 14–17). This will further exacerbate the dramatic consequences of livestock production for various environmental problems from climate change to soil degradation to biodiversity (Benton et al. 2021).

The growing availability and affordability of meat became possible only through a profound transformation of livestock breeding and meat production. Scientific and technical developments like the use of more productive breeds, increased grain feeding or the administration of antibiotics made it possible to increase both the rate and density of livestock production (FAO 2006, pp. 11–14). Also, over the last decades the average size of the breeding and processing facilities grew sharply (Fitzgerald 2010, p. 63). The slaughterhouse, for example, emerged in the early 19th century as a centralized institution for the killing of livestock. In the second half of the century, industrialized meat-processing developed in urban areas like the Union Stock Yard in Chicago—“a massive slaughterhouse complex unlike anything that had come before it” (Fitzgerald 2010, p. 60). Later, from the 1960s on, the “modern, high-volume, slaughterhouses located closer to the supply of livestock” developed in step with the emergence and intensification of factory farming (Fitzgerald 2010, p. 63).

The concentration and intensification of meat production is often interpreted as a quasi-automatic result of logistic and technological developments (like the implementation of refrigeration or mechanization) or economic principles (like economies of scale). In contrast, historical and sociological perspectives also highlight social and political reasons like the undermining of labor unions or low standards of worker protection (Fitzgerald 2010, pp. 61–62). In her article in this issue, *Katrin Hirte *contributes to a more comprehensive understanding of the transformation of meat production. Hirte’s analysis addresses the dynamics behind the concentration processes in the German slaughterhouse industry. As she demonstrates, these concentration processes are predominantly interpreted within the framework of agricultural economics as the necessary outcome of economies of scale. By referring to the example of the “Böckenhoff plan,” which served as a kind of masterplan for the concentration of the slaughtering industry in the new German states after 1989, Hirte shows how problematic such a narrow economic explanation is: First, because it ignores the political decision-making processes behind the transformation of the slaughterhouse industry; second, because it conceals the decisive performative role the discipline of agricultural economics itself has played in the shaping of the German meat industry. This failure of agricultural economics to reflect its own market-centered perspective, Hirte argues, continues to hamper a proper understanding of various problematic consequences of meat production.

## Dissonance: the struggle over meat

One of the most striking characteristics of human-animal relations in modern Western societies is that the volume of meat production skyrocketed since the beginning of industrialization while simultaneously the sensitivities to the suffering of animals increased significantly. Hence, there exists a pronounced dissonance between attitudes and sensitivities on the one side and meat production and consumption patterns on the other hand. In social psychology, various studies have analyzed this tension under the heading of the “meat paradox” (Bastian and Loughnan 2017; Loughnan, Haslam, and Bastian 2010; Piazza et al. 2015). Using elaborate experimental designs, these studies identify different strategies that help individuals to overcome the cognitive dissonances caused by eating meat. Examples for such strategies are the rationalizing of meat consumption as normal, necessary or natural or by denying mental states or pain to animals. Although they produce crucial experimental insights, Frithjof Nungesser argues (2020), the psychological approach to the “meat paradox” is limited in at least three respects.

First, the psychological literature usually presumes that meat consumption causes cognitive dissonance and then focusses on the strategies that are used to eliminate it. Little attention is paid to the *preconditions for the emergence of this dissonance*. The perception of certain animals as sentient and morally relevant beings is not a cross-cultural universal but needs to be explained with reference to broader sociohistorical processes. To explain the growing sensitivity to the welfare of animals, several sociological studies draw on Norbert Elias’s theory of the civilization process (Fiddes 1991, ch. 7; Jasper 2008, pp. 162–163; Pachirat 2011, pp. 9–11, 249–251; Traïni 2016; Witte 2019). Referring to Elias’s theory makes it possible to trace back changes in the human-animal relationship to a broad spectrum of historical macro- and microsocial transformations such as the monopolization of force by the state which coevolves with an increase in the affective aversion against violence in general.

Second, in contrast to psychological studies, sociological approaches show that *meat-induced dissonance is an object of social struggles*. Experiences of dissonance are not merely a result of individual behavior and cognition but are mediated by debates, criticisms and protests that articulate and amplify the increased sensitivity to animal suffering. This holds especially true for the different social movements that are connected to meat consumption (Jasper 2008, p. 156). Almost from its beginning, the mass-production of meat was met with criticism, which developed—broadly speaking—in three waves: First, in the second half of the 19th century, the first animal-protection laws were passed and the first animal protection societies were founded (Spencer 2002, ch. 11; Traïni 2016, ch. 1). Second, vegetarianism emerged as a “cultural movement” (Cherry 2006) or “life-style movement” (Haenfler, Johnson, and Jones 2012).^6^ While animal protection campaigns mostly try to prevent cruelty to animals and to improve the conditions of livestock production, vegetarianism challenges the legitimacy of meat consumption in general. Such demands were made in various countries such as France and England as early as the end of the 18th century in specific milieus which were culturally and politically connected to the revolutionary currents of their time. In Germany, the *Lebensreform *movement contested the norm to eat meat and promoted vegetarianism. German sociologist Eva Barlösius ([1999] 2011, p. 118) describes this as a “countercultural and anti-hierarchic protest.” A few decades after the first animal protection legislation, the criticism of meat production and consumption was then institutionalized in the form of vegetarian societies and associations (Stuart 2007, ch. 21, 23). Third, starting in the 1940s, parts of the British vegetarian movement became more radical and demanded the end of all animal use, thus giving rise to the first vegan society (Leneman 1999). Since the 1970s, this rigorous criticism of animal use became more and more vocal in the form of the animal rights movement (Jasper and Nelkin 1992). Nowadays in many Western societies, a remarkable trend towards meat-free diets can be observed. Today, perhaps more than ever, meat consumption and its consequences are objects of heated public debates (Witte 2019). This trend also led to backlashes such as the “new carnivore movement,” which is also connected to other cultural debates and concepts such as masculinity (Gutjahr 2013; Parry 2010). Conflicts about meat consumption can thus also be analyzed as gendered conflicts: While veganism is strategically connected to a muscular masculinity (Winter 2019), the defense of meat also refers to strong images of masculinity.

Third, sociological studies emphasize that the avoidance of meat-induced dissonance is not just a matter of individual psychological processes. Instead, this *avoidance is situated in specific infrastructures and discourses of society*. The most obvious materialization of this socially facilitated avoidance of meat-related dissonance is the isolation of the killing and processing of animals from the public. It is again Elias, who describes how this isolation developed over the centuries: “From a standard of feeling by which the sight and carving of a dead animal on the table are actually experienced as pleasurable, or at least as not at all unpleasant, the development leads to another standard by which reminders that the meat dish has something to do with the killing of an animal are avoided to the utmost.” (Elias [1939] 2000, p. 102) This “civilizing” of eating meat also involves the relocation of dealing with animal flesh to “specialized enclaves” (Elias [1939] 2000, p. 106) remote from the eating table, especially to slaughterhouses. As Timothy Pachirat (2011, ch. 1) argues in his important ethnography, industrialized slaughter today is “hidden in plain sight.” Slaughterhouses are usually embedded in industrial areas and do not differ significantly from other buildings. This perceptual isolation of slaughterhouses is not only attained by physical distance and material barriers that minimize the visual, auditory or olfactory contact with the animals which are killed and processed for food. Moreover, contact is avoided by the temporal and material organization of animal transports or by the social isolation of the people employed in the slaughterhouses. The social avoidance of animal use and “dirty work,” it seems, also contributes to the disregard of the precarious working conditions in slaughterhouses (Voivozeanu 2019), which briefly attracted attention in the course of the coronavirus pandemic in different countries because slaughterhouses and processing plants became hotspots of infection.

If one connects the increasing sensitivity to animal welfare, the increased criticism of animal use and the perceptual and social isolation of meat production, it becomes obvious that workers in the meat industry find themselves in a challenging social position. In his contribution to this issue, *Marcel Sebastian* takes his start from this situation. Based on thirteen problem-centered interviews with employees of six slaughterhouses in Germany, Sebastian studies how slaughterhouse workers cope with their moral stigmatization and how their coping strategies are connected to cultural discourses about animal welfare and meat production. As his analysis shows, the employees deal with their moral stigmatization by rejecting the cultural ideas that underlie the criticisms and by arguing that outsiders lack the knowledge necessary for a valid assessment of their work.

## Outlook

The meat-society nexus is constantly changing and evolving. The described dynamics of distinction, domination and dissonance are currently accelerating and feedback effects can increasingly be observed. Not only is the role of meat in common diets changing as society changes, also effects of the industrial production of meat on the environment and animals have their influence on social transformations. Technological innovations, like in-vitro meat or insects as protein sources are recognizable on the food horizon. However, these products rely on the acceptance of consumers and far-reaching changes of eating cultures in Western societies. To deal with the social problems related to meat, further sociological reflection and research is necessary and it is already foreseeable that this topic will gain importance in the near future. With this issue in hand, we want to make an insightful contribution not only to the sociology of food, eating and human-animal relations. We are convinced that the topics assembled in this issue are relevant to sociological research in general. Last but not least, we want to thank the authors for their valuable articles and the reviewers for sharing their expertise by commenting on the manuscripts.

## Footnotes

1 There is a debate in nutritional science on the scope of the discipline. Some authors demand a more holistic approach that includes biological, social and ecological aspects of nutrition (e.g. the “Gießen Declaration”: Leitzmann and Cannon 2006).

2 These changes have not been limited to *what* people eat but also to *how* they eat. Eating out, to-go-food (not only in the current pandemic), and convenience food change eating practices fundamentally. On the one hand, this means that traditional settings, such as the family meal decline (Schönberger and Methfessel 2011, pp. 7–8). On the other hand, the trend to eat in public amplifies the importance of symbolic representations of food (Rückert-John et al. 2011, pp. 42–43).

3 Quotes from German-speaking literature are translated by the authors.

4 In an analysis of representative survey-data, Einhorn (2020, p. 49) presents remarkable statistical insights into contemporary meat-eating habits. As she shows, meat consumption decreases with higher levels of cultural capital (in the form of formal education).

5 See the organization’s statistical website for details (www.fao.org/faostat).

6 Importantly, vegetarianism is not a homogenous movement. From early on there were two major strands within the vegetarian movement that can still be distinguished today: One strand criticizes meat consumption as unnatural and unhealthy for humans, while the other strand emphasizes that meat (and other animal products) causes animal suffering (Leneman 1999). Therefore, only certain strands of vegetarianism can be interpreted as “lifestyle politics” (Micheletti and Stolle 2011).
